# PROPTIMUS LIVE: local constrained α-carbon optimization of proteins

**DOI:** 10.1093/nar/gkag511

**Published:** 2026-05-20

**Authors:** Tomáš Svoboda, Michal Mikuš, Lukáš Bohuš, Tomáš Raček, Gabriela Bučeková, Dominik Tichý, Karel Berka, Radka Svobodová, Ondřej Schindler

**Affiliations:** National Centre for Biomolecular Research, Faculty of Science, Masaryk University, Kamenice 5, Brno 625 00, Czech Republic; CEITEC – Central European Institute of Technology, Masaryk University, Kamenice 5, Brno 625 00, Czech Republic; Institute of Computer Science, Masaryk University, Šumavská 525/33, Brno 602 00, Czech Republic; National Centre for Biomolecular Research, Faculty of Science, Masaryk University, Kamenice 5, Brno 625 00, Czech Republic; National Centre for Biomolecular Research, Faculty of Science, Masaryk University, Kamenice 5, Brno 625 00, Czech Republic; CEITEC – Central European Institute of Technology, Masaryk University, Kamenice 5, Brno 625 00, Czech Republic; National Centre for Biomolecular Research, Faculty of Science, Masaryk University, Kamenice 5, Brno 625 00, Czech Republic; CEITEC – Central European Institute of Technology, Masaryk University, Kamenice 5, Brno 625 00, Czech Republic; National Centre for Biomolecular Research, Faculty of Science, Masaryk University, Kamenice 5, Brno 625 00, Czech Republic; Department of Physical Chemistry, Faculty of Science, Palacký University, tř. 17. listopadu 12, Olomouc 771 46, Czech Republic; National Centre for Biomolecular Research, Faculty of Science, Masaryk University, Kamenice 5, Brno 625 00, Czech Republic; CEITEC – Central European Institute of Technology, Masaryk University, Kamenice 5, Brno 625 00, Czech Republic; National Centre for Biomolecular Research, Faculty of Science, Masaryk University, Kamenice 5, Brno 625 00, Czech Republic; CEITEC – Central European Institute of Technology, Masaryk University, Kamenice 5, Brno 625 00, Czech Republic

## Abstract

High-quality protein structures are essential for a wide range of computational chemistry applications. While experimental methods and predictive algorithms provide high-accuracy positions of protein residues relative to one another, the local quality of these structures, including bond lengths, angles, and individual atom positions, often lacks the same level of precision. To address this, we developed PROPTIMUS LIVE, a web application offering local constrained α-carbon optimization of protein structures. PROPTIMUS LIVE is powered by the QM-accurate, physics-based GFN-Force-Field and accelerated using a divide-and-conquer approach, allowing typical optimizations to finish within minutes. Optimized structures can be visualized and investigated directly via the integrated Mol* Viewer. The service is freely available at https://proptimus.ceitec.cz/live with no login required, including for commercial use.

## Graphical Abstract

**Graphical Abstract:**
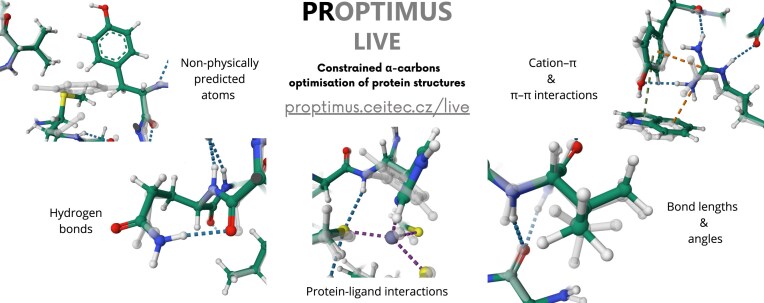

## Introduction

The three-dimensional (3D) structure is the crucial factor that determines the interactions and the function of a protein [1]. Protein structures can be obtained either experimentally or through prediction algorithms. The most widely utilized repositories for protein structures are the Protein Data Bank (PDB) [2] and the AlphaFold Protein Structure Database (AlphaFold DB) [3]; the PDB contains over 250 000 experimental structures, while AlphaFold DB provides access to >200 million machine-learning (ML) predicted structures. However, these structures may contain various local issues, such as unusual bond lengths and angles, or missing hydrogen bonds and other inter-residual interactions [4–6]. In experimentally determined structures, such inaccuracies are typically a consequence of the limited precision of the underlying experimental data (e.g. low resolution in the case of X-ray crystallography) [7]. In ML-predicted structures, such inaccuracies stem from the focus of prediction algorithms on $\mathrm{C}_\alpha$ coordinates; thus, the positioning of the remaining atoms, particularly in the side chains, tends to be less accurate [8].

The quality of interatomic and inter-residue characteristics is critical for achieving accurate results in various applications of computational chemistry [4]. Applications sensitive to structure quality include virtual screening [9], molecular docking [10], QSPR modelling [11], partial atomic charge calculations [12], calculations using semiempirical or QM/MM methods [13], or training ML models [14]. For this reason, protein structures are usually optimized before further research [4]. To avoid extensive deviations from the original structure and to ensure that modifications focus only on local characteristics and potential structural inconsistencies, specific constraints are applied during the optimization process, most commonly to $C_\alpha$ coordinates [4]. For optimization of protein structures, force-field methods [4,15] are commonly used.

Protein optimization can be performed locally; however, it often requires specialized software and IT expertise. Web-based services therefore provide convenient and accessible alternatives for many users. Examples of available web applications include GalaxyRefine [16], GalaxyRefine2 [17], 3Drefine [18], ModRefiner [19], YASARA Energy Minimization Server [20], or Rosetta Relax Server [21]. These applications optimize protein structures using various sampling techniques such as conformational search, simulated annealing, or molecular dynamics. The energy of the highest-ranking samples is then locally minimized. Furthermore, all these applications rely on knowledge-based force fields, which can be interpreted as parametrized statistical potentials [22]. Due to their computational efficiency, these force fields are particularly well-suited as scoring functions for evaluating large volumes of samples. However, they do not describe atomic and inter-residue interactions as accurately as physics-based force fields. Specifically, whereas empirical functions rely on statistical patterns from known structures, physics-based methods explicitly model the underlying physical forces, such as electrostatics and van der Waals interactions [23].

This motivation led us to develop the PROPTIMUS LIVE web application, providing local optimization of protein structures with constrained $C_\alpha$. PROPTIMUS LIVE is powered by the QM-accurate, physics-based GFN-Force-Field (GFN-FF) [15], accelerated by the divide-and-conquer RAPHAN approach [24]. GFN-FF is fully generic, enabling PROPTIMUS LIVE to optimize proteins and their ligands. The main goal of PROPTIMUS LIVE is to optimize bond lengths and angles, as well as inter-residue interactions, including hydrogen bonds, cation–$\pi$ interactions, and $\pi$–$\pi$ stacking. The results are visualized using the Mol* Viewer software [25], enabling users to analyse the optimized structure directly within the application. It is important to note that PROPTIMUS LIVE is not intended as an alternative to other web applications for protein structure optimization, given that it does not perform the often-required conformational sampling and focuses solely on accurate local optimization. Instead, PROPTIMUS LIVE serves as a complementary tool that brings QM accuracy to knowledge-based force-field-optimized structures, as already has been advised [23].

## Description of the web server

### Optimization method

Protein structures are optimized using the GFN-FF force field [15] from the xtb software [26]. The solvation of protein structures is included using the analytical linearized Poisson-Boltzmann (ALPB) implicit solvent model [27]. Geometry optimization is performed using the ANCopt optimization engine from xtb software to identify local energy minima, corresponding to 0 K energy minimizations without thermal sampling. The optimization is strictly deterministic, ensuring that repeated runs with identical input produce the same output. The GFN-FF force field is employed as the sole energy function during structural minimization, accounting for all intra- and inter-residue interactions.

Optimization of protein structure is further accelerated by the divide-and-conquer RAPHAN approach, a faster alternative to constrained $\mathrm{C}_\alpha$ optimization. In this iterative approach, the entire structure is not optimized at once. Instead of this, in each iteration for all residues, a substructure consisting of that residue and its surrounding atoms is created, and each substructure is optimized separately, which leads to a rapid acceleration of calculation while maintaining high precision. A detailed description of RAPHAN, along with extensive performance benchmarks, can be found in [24].

### The PROPTIMUS LIVE workflow

One of the major design decisions for PROPTIMUS LIVE was to provide the user with an intuitive interface that would facilitate protein structure optimization without requiring the user to configure all process parameters. The workflow can be described in five steps:

#### 1. Input

The PROPTIMUS LIVE application supports three input options. The first option is to upload a PDB file. The second option is to enter a four-character PDB ID, and the protein is then downloaded from PDB. The third option is to enter a UniProt accession number, and the protein is downloaded from AlphaFold DB. For each optimization of the structure, the pH value can be specified, with a default setting of 7.

#### 2. Preprocessing

**Figure 1.**
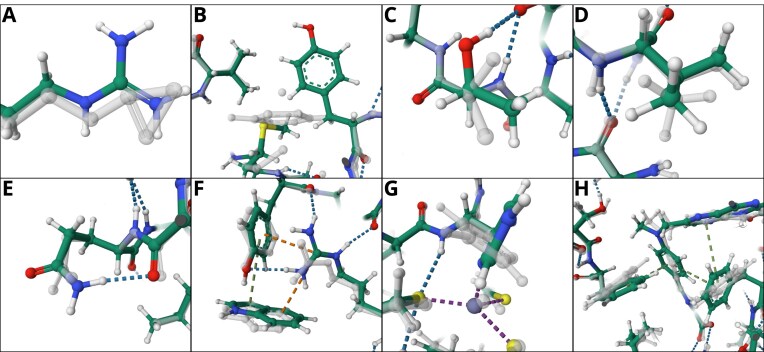
Visualization of original (grey) and optimized structures (coloured) using Mol* Viewer. (**A**) Correcting the non-physically positioned atoms in ARG22 in the structure with UniProt ID: A4QJE9, (**B**) correcting non-physically positioned atoms in residues TYR22 and MET26 in the structure with UniProt ID Q9RS06, (**C**) optimization of the eclipsed dihedral angle of THR555 in the structure with UniProt ID Q57N56, (**D**) optimization of the eclipsed dihedral angle of VAL123 in the structure with UniProt ID: Q3M859, (**E**) formation of a hydrogen bond between ARG369 and GLN370 in the structure with UniProt ID B7ZW16, (**F**) formation of a cation–$\pi$ interaction of ARG237 in the structure with UniProt ID: Q3M859, (**G**) optimization of HIS 25 to achieve a coplanar orientation with the zinc ion in the structure with PDB ID: 5xht, (**H**) formation of a $\pi$–$\pi$ interaction of MTX170 in the structure with PDB ID: 1ao8. Structures A–F were sourced from the AlphaFold DB, whereas structures G–H were obtained from the PDB.

If hydrogen atoms are missing in the protein, the structure undergoes preprocessing, which comprises two distinct phases:

**(i) Correction of unphysically positioned atoms:** In the case of ML-predicted structures from AlphaFold DB, non-physically positioned atoms are detected, removed, and subsequently re-added (shown in Fig. 1A and B). Atoms in unphysical positions are identified based on the discrepancy between the set of canonical bonds as defined in Chemical Compound Dictionary [28] and those inferred by RDKit [29]. Removal and re-addition are performed using the Biopython [30] and PDB2PQR [31] libraries.

**(ii) Adding hydrogens:** Hydrogen atoms are added to the structure based on the user-defined pH. For proteins consisting of standard amino acids, the pdb2pqr software [31] is employed alongside the PROPKA3 library [32], which considers the influence of the surrounding environment when determining the protonation states of titratable residues. If the protein includes non-standard residues, PROPTIMUS LIVE utilizes a more universal hydride library [33]; however, this library determines the protonation states of titratable amino acids solely based on their dissociation constants.

#### 3. Structure optimization

The structure is locally optimized using GFN-FF with constrained $C_\alpha$ atoms [15]. Acceleration via the RAPHAN approach [24], combined with parallelization, enables the optimization of an average protein structure within minutes. For proteins containing only standard amino acids, the process proceeds at an average rate of 1000 atoms per minute. If a protein contains non-standard residues, the calculation takes approximately twice as long, as no atoms are constrained within these residues.

#### 4. Visualization of the optimized protein structure with Mol*

Calculation results are available on the Results page, where the central component is the embedded Mol* Viewer. The default view displays the original structure in translucent grey, overlaid on the optimized structure, with atoms coloured by element. The translucent grey background structure can be replaced with the preprocessed structure (if the structure originates from the AlphaFold DB and has undergone preprocessing), the optimization trajectory, or removed entirely to visualize only the optimized structure. Atoms in the optimized structure can also be colour-coded by the magnitude of their displacement during optimization.

Three tables with calculation logs are accessible to the user via the Results page. The first table contains information on residues that contained non-physically positioned atoms, along with whether these atoms were successfully repaired during preprocessing. The second table lists residues that failed to converge during optimization (see Limitations). The third table lists inter-residue interactions that were formed or broken during the optimization process. Users can zoom in on respective residues in the Mol* Viewer through links in the table. Inter-residue interactions are detected after optimization by Biotite library [34].

#### 5. Download of the optimized structure

The results can also be downloaded and used for further processing. PROPTIMUS LIVE includes the input structure, preprocessed structure, optimization trajectory, and optimized structure in the PDB format, all in a downloadable ZIP file for user convenience.

### Limitations

PROPTIMUS LIVE is limited to the PDB format because the xtb software, which provides GFN-FF, does not support mmCIF. If a PDB file contains multiple models, only the first one is optimized. The input protein structure must be reasonably close to a “normal” chemical-bonding situation such that the GFN-FF initial topology analysis works properly [15]. Optimization may encounter convergence issues, particularly in protein–ligand optimizations where no atoms are constrained, leading to a higher number of degrees of freedom. If the optimization fails to converge for certain residues, the user is informed on the Results page.

## Results and discussion

PROPTIMUS LIVE optimizes particularly atomic and inter-residue interactions. For demonstration purposes, we present eight examples which are also available on the PROPTIMUS LIVE webpage.

During preprocessing, non-physically positioned atoms are corrected as illustrated in Fig. 1A and B. Improving dihedral angles from eclipse conformations are shown in Fig. 1C and D. Formation of inter-residue interactions, including hydrogen bond and cation–$\pi$ interactions, is displayed in Fig. 1E and F. Optimizations of interaction between ligand and protein are demonstrated in Fig. 1G and H.

### Performance of web server

The PROPTIMUS LIVE server is based on the GFN-FF force field, which has been previously validated for accuracy on X-ray crystallography structures [15]. Computational efficiency is ensured by the RAPHAN acceleration approach, which has been demonstrated to produce optimized structures comparable to those obtained via GFN-FF optimization with $\mathrm{C}_\alpha$ constraints [24]. Given that the refinement category is no longer included in the Critical Assessment of Structure Prediction (CASP) challenges [35], we evaluated PROPTIMUS LIVE’s performance against several established web-based refinement tools: GalaxyRefine [16], GalaxyRefine2 [17], ModRefiner [19], and Rosetta Relax Server [21]. Evaluation of the refined models focused on the number of detected inter-residue interactions. Notably, in structures optimized by PROPTIMUS LIVE, the greatest number of these interactions across the test set among all tested servers. Detailed benchmarking data and comparative metrics are available in the Supplementary Files.

To evaluate PROPTIMUS LIVE, we further investigated how protein structure quality impacts partial atomic charges. We compared charges from original AlphaFold DB structures against those refined by PROPTIMUS LIVE and GFN1-xTB. Notably, the charge deviation between structures optimized by PROPTIMUS LIVE and GFN1-xTB was found to be three times smaller than the deviation between the original structures and those optimized by GFN1-xTB. These findings demonstrate that PROPTIMUS LIVE optimization leads to a more realistic description of the electrostatics profile. Detailed benchmarking data are provided in the Supplementary Files.

## Conclusion

In this article, we present PROPTIMUS LIVE, a web application for fast and accurate local optimization of protein structures. The server uses the physics-based, generic GFN-FF force field accelerated by the divide-and-conquer RAPHAN approach. Optimized structures can be visualized and explored directly in the web interface using the Mol* Viewer. PROPTIMUS LIVE therefore provides an accessible tool for improving local structural geometry prior to downstream computational analyses.

## Data Availability

PROPTIMUS LIVE is freely available to anyone at https://proptimus.ceitec.cz/live with no login requirement. The source codes are available at https://github.com/sb-ncbr/proptimus_live under the MIT licence. They are also archived in the OneData repository at https://doi.org/10.58074/93w8-9226 together with results of PROPTIMUS LIVE testing.

## References

[1] Thompson MC, Yeates TO, Rodriguez JA. Advances in methods for atomic resolution macromolecular structure determination. F1000Research. 2020;9:667. 10.12688/f1000research.25097.1 PMC7333361 32676184

[2] Burley SK, Bhatt R, Bhikadiya C et al. Updated resources for exploring experimentally-determined PDB structures and computed structure models at the RCSB Protein Data Bank. Nucleic Acids Res. 2025;53:D564–74. 10.1093/nar/gkae1091 39607707 PMC11701563

[3] Varadi M, Anyango S, Deshpande M et al. AlphaFold Protein Structure Database: massively expanding the structural coverage of protein-sequence space with high-accuracy models. Nucleic Acids Res. 2022;50:D439–44. 10.1093/nar/gkab1061 34791371 PMC8728224

[4] Adiyaman R, McGuffin LJ. Methods for the refinement of protein structure 3D models. Int J Mol Sci. 2019;20:2301. 10.3390/ijms20092301 31075942 PMC6539982

[5] Smart OS, Horský V, Gore S et al. Worldwide Protein Data Bank validation information: usage and trends. Biol Crystallogr. 2018;74:237–44. 10.1107/S2059798318003303 PMC5947764 29533231

[6] Terwilliger TC, Liebschner D, Croll TI et al. AlphaFold predictions are valuable hypotheses and accelerate but do not replace experimental structure determination. Nat Methods. 2024;21:110–6. 10.1038/s41592-023-02087-4 38036854 PMC10776388

[7] Bučeková G, Doshchenko V, Svoboda T et al. Analysis of cyclohexane, cyclopentane, and benzene conformations in ligands for PDB X-ray structures using the Hill-Reilly approach. J Cheminform. 2026;18:17. 10.1186/s13321-026-01154-0 41519762 PMC12882122

[8] Jumper J, Evans R, Pritzel A et al. Highly accurate protein structure prediction with AlphaFold. Nature. 2021;596:583–9. 10.1038/s41586-021-03819-2 34265844 PMC8371605

[9] Madhavi Sastry G, Adzhigirey M, Day T et al. Protein and ligand preparation: parameters, protocols, and influence on virtual screening enrichments. J Comput-Aided Mol Des. 2013;27:221–34. 10.1007/s10822-013-9644-8 23579614

[10] Scardino V, Di Filippo JI, Cavasotto CN. How good are AlphaFold models for docking-based virtual screening?. iScience. 2023;26:105920. 10.1016/j.isci.2022.105920 36686396 PMC9852548

[11] Geidl S, Svobodová Varřková R, Bendová V et al. How does the methodology of 3D structure preparation influence the quality of pKa prediction?. J Chem Inf Model. 2015;55:1088–97. 10.1021/ci500758w 26010215 PMC5098400

[12] Bleiziffer P, Schaller K, Riniker S. Machine learning of partial charges derived from high-quality quantum-mechanical calculations. J Chem Inf Model. 2018;58:579–90. 10.1021/acs.jcim.7b00663 29461814

[13] Bursch M, Mewes JM, Hansen A et al. Best-practice DFT protocols for basic molecular computational chemistry. Angew Chem Int Edit. 2022;61:e202205735. 10.1002/anie.202205735 PMC9826355 36103607

[14] Kaptan S, Vattulainen I. Machine learning in the analysis of biomolecular simulations. Adv Phys: X. 2022;7:2006080. 10.1080/23746149.2021.2006080

[15] Spicher S, Grimme S. Robust atomistic modeling of materials, organometallic, and biochemical systems. Angew Chem Int Edit. 2020;59:15665–73. 10.1002/anie.202004239 PMC7267649 32343883

[16] Heo L, Park H, Seok C. GalaxyRefine: protein structure refinement driven by side-chain repacking. Nucleic Acids Res. 2013;41:W384–8. 10.1093/nar/gkt458 23737448 PMC3692086

[17] Lee GR, Won J, Heo L et al. GalaxyRefine2: simultaneous refinement of inaccurate local regions and overall protein structure. Nucleic Acids Res. 2019;47:W451–5. 10.1093/nar/gkz288 31001635 PMC6602442

[18] Bhattacharya D, Nowotny J, Cao R et al. 3Drefine: an interactive web server for efficient protein structure refinement. Nucleic Acids Res. 2016;44:W406–9. 10.1093/nar/gkw336 27131371 PMC4987902

[19] Xu D, Zhang Y. Improving the physical realism and structural accuracy of protein models by a two-step atomic-level energy minimization. Biophys J. 2011;101:2525–34. 10.1016/j.bpj.2011.10.024 22098752 PMC3218324

[20] Krieger E, Joo K, Lee J et al. Improving physical realism, stereochemistry, and side-chain accuracy in homology modeling: four approaches that performed well in CASP8. Proteins. 2009;77:114–22. 10.1002/prot.22570 19768677 PMC2922016

[21] Lyskov S, Chou FC, Conchuir SO et al. Serverification of molecular modeling applications: the Rosetta Online server that includes everyone (ROSIE). PloS One. 2013;8:e63906. 10.1371/journal.pone.0063906 23717507 PMC3661552

[22] Thomas PD, Dill KA. Statistical potentials extracted from protein structures: how accurate are they?. J Mol Biol. 1996;257:457–69. 10.1006/jmbi.1996.0175 8609636

[23] Lam JH, Katritch V. Navigating structure-based drug discovery with emerging innovations in physics-and knowledge-based approaches. npj Drug Disc. 2025;2:29. 10.1038/s44386-025-00031-4 PMC12669043 41341471

[24] Schindler O, Bučeková G, Svoboda T et al. Per-residue optimisation of protein structures: rapid alternative to optimisation with constrained alpha carbons. bioRxiv, 10.1101/2025.11.24.690085, 26 November 2025, preprint: not peer reviewed.

[25] Sehnal D, Bittrich S, Deshpande M et al. Mol* Viewer: modern web app for 3D visualization and analysis of large biomolecular structures. Nucleic Acids Res. 2021;49:W431–7. 10.1093/nar/gkab314 33956157 PMC8262734

[26] Bannwarth C, Caldeweyher E, Ehlert S et al. Extended tight-binding quantum chemistry methods. Wiley Interdiscipl Rev Comput Mol Sci. 2021;11:e1493. 10.1002/wcms.1493

[27] Ehlert S, Stahn M, Spicher S et al. Robust and efficient implicit solvation model for fast semiempirical methods. J Chem Theory Comput. 2021;17:4250–61. 10.1021/acs.jctc.1c00471 34185531

[28] Berman H, Henrick K, Nakamura H. Announcing the worldwide protein data bank. Nat Struct Mol Biol. 2003;10:980. 10.1038/nsb1203-980 14634627

[29] RDKit: Open-source cheminformatics. 10.5281/zenodo.591637

[30] Cock PJ, Antao T, Chang JT et al. Biopython: freely available Python tools for computational molecular biology and bioinformatics. Bioinformatics. 2009;25:1422. 10.1093/bioinformatics/btp163 19304878 PMC2682512

[31] Dolinsky TJ, Nielsen JE, McCammon JA et al. PDB2PQR: an automated pipeline for the setup of Poisson–Boltzmann electrostatics calculations. Nucleic Acids Res. 2004;32:W665–7. 10.1093/nar/gkh381 15215472 PMC441519

[32] Olsson MH, Søndergaard CR, Rostkowski M et al. PROPKA3: consistent treatment of internal and surface residues in empirical pKa predictions. J Chem Theory Comput. 2011;7:525–37. 10.1021/ct100578z 26596171

[33] Kunzmann P, Anter JM, Hamacher K. Adding hydrogen atoms to molecular models via fragment superimposition. Algorithm Mol Biol. 2022;17:7. 10.1186/s13015-022-00215-x PMC8966362 35351165

[34] Kunzmann P, Müller TD, Greil M et al. Biotite: new tools for a versatile Python bioinformatics library. BMC bioinformatics. 2023;24:236. 10.1186/s12859-023-05345-6 37277726 PMC10243083

[35] Kryshtafovych A, Schwede T, Topf M et al. Critical assessment of methods of protein structure prediction (CASP)—Round XV. Proteins. 2023;91:1539–49. 10.1002/prot.26617 37920879 PMC10843301

